# Effects of ayahuasca on mental health and quality of life in naïve users: A longitudinal and cross-sectional study combination

**DOI:** 10.1038/s41598-020-61169-x

**Published:** 2020-03-05

**Authors:** Daniel F. Jiménez-Garrido, María Gómez-Sousa, Genís Ona, Rafael G. Dos Santos, Jaime E. C. Hallak, Miguel Ángel Alcázar-Córcoles, José Carlos Bouso

**Affiliations:** 1 ICEERS – International Center for Ethnobotanical Education, Research, and Services, Barcelona, Spain; 2 Universitat Rovira i Virgili, Department of Anthropology, Philosophy and Social Work, Tarragona, Spain; 3 Department of Neuroscience and Behavior, Ribeirão Preto Medical School, University of São Paulo, São Paulo, SP Brazil; 4 National Institute for Translational Medicine (INCT-TM), CNPq, São Paulo, Brazil; 5 Department of Biological & Health Psychology, School of Psychology, Madrid Autonomous University, 28049 Madrid, Spain

**Keywords:** Quality of life, Epidemiology

## Abstract

Ayahuasca is a hallucinogenic decoction used as a traditional medicine in several Amazonian regions. The ritualistic use of ayahuasca has spread throughout many countries, making it necessary to study its risks and benefits. Two sub-studies were designed for this investigation. In sub-study 1, a psychiatric interview and a battery of questionnaires were administered to subjects (n = 40) before their first ayahuasca use. Two follow-ups were conducted at 1 and 6 months. In sub-study 2, the same interview and battery of questionnaires were administered to long-term ayahuasca users (n = 23) and their scores were compared with those of the ayahuasca-naïve group. In the first assessment, nearly half (45%) of the naïve users were found to meet the diagnostic criteria for a psychiatric disorder. After the ayahuasca use, more than 80% of those subjects showed clinical improvements that persisted at 6 months. The questionnaires showed significant reductions in depression and psychopathology. Regarding sub-study 2, long-term users showed lower depression scores, and higher scores for self-transcendence and quality of life, as compared to their peers in sub-study 1. Further controlled and observational naturalistic studies assessing the eventual risks and potential benefits of ayahuasca are warranted.

## Introduction

Ayahuasca is a psychoactive beverage that results from the decoction of *Banisteriopsis caapi* and *Psychotria viridis*, plants rich in β-carbolines (harmine or tetrahydroharmine, among others) and N,N-dimethyltryptamine (DMT), respectively. DMT is a partial agonist of serotonin (5-HT) receptors^1^, but it can interact with other receptors as well (for a review see Carbonaro & Gatch^2^). The hallucinogenic effects are primarily caused by the combination of the monoamine-oxidase A (MAO-A) inhibiting properties of β-carbolines and DMT, which results in the oral bioavailability of the latter^3^.

Ayahuasca has been traditionally used in several communities of the Amazonia, but in recent decades its use has spread throughout the world^4^, first to urban areas of Brazil, where syncretic religions such as Santo Daime, União do Vegetal and Barquinha were established^5^, and then to other contexts, including several countries of the world where ayahuasca retreat centers have been developed and/or neoshamanic groups exist^6,7^.

Concurrent with this increased public interest in ayahuasca ceremonies, there has been major interest from the academic and biomedical fields regarding its potential health effects^8,9^. Data from observational studies suggest that ayahuasca and its active ingredient DMT may have anxiolytic properties^10,11^. Furthermore, it has not been associated with increased psychopathology or with impairments in neuropsychological functioning^12,13^. An open-label clinical study found significant therapeutic benefits among patients with treatment-resistant major depressive disorder (MDD) after the administration of a single dose of ayahuasca^14,15^. Additionally, one randomized, placebo-controlled clinical trial was recently published, showing that, compared to placebo, a single ayahuasca dose was associated with significant reductions in depressive symptoms in MDD patients^16^.

The mechanisms through which ayahuasca produces therapeutic effects are not completely understood. First, DMT is widely found in plants and mammals, including humans^17^. It acts as a partial agonist at 5-HT receptors, and several studies have shown that the 5-HT_2A_ receptor site could be the main target^1^. Additionally, neuroimaging studies (described below) show that the neural effects of ayahuasca, in both healthy and depressive subjects, are mediated by brain areas rich in 5-HT_2A_ receptors. As far as β-carbolines concerned, they also show potential neuroprotective effects besides their MAO-inhibiting properties^18^.

Beyond the complex effects the isolated compounds have on different neurotransmitter systems, there are neuroimaging studies that can help clarify ayahuasca’s potential mechanisms of action. Single photon emission computed tomography (SPECT) studies reported that ayahuasca increases blood perfusion in frontal brain regions, the insula, the left nucleus accumbens, the left amygdala, the parahippocampal gyrus, and the left subgenual area^15,19^. This pattern suggests that ayahuasca’s effects are related to introspection and emotional processing. Studies using functional magnetic resonance imaging (fMRI) have observed activations in the occipital, temporal, and frontal areas of the brain. Remarkably, even with eyes closed, levels of activation in the occipital area were consistent with the visionary experience^20^. Ayahuasca also activates the frontal cortex and areas involved in episodic memory. Furthermore, in one study using MRI, an inverse correlation between cortical thickness in the posterior cingulate cortex (PCC) and intensity and duration of previous ayahuasca use was observed^13^. This is highly relevant due to the direct implication of PCC in the default mode network (DMN), and it suggests that regular ayahuasca use could potentially lead to structural changes in certain brain areas. Evidence of decreased DMN activity after ayahuasca use supports this finding^21^.

Due to the current crisis in psychopharmacology^22^ and the lack of effective medications for the treatment of psychological or neurological disorders^23^, alongside the continued exoticization of indigenous cultures, the number of people attracted by alternative medical practices such as the ritualistic use of ayahuasca has been increasing^4^. Given this context, the effects of ayahuasca should be assessed especially in those people who have no previous experience with the decoction. This would help to avoid the bias present in retrospective observational studies for which only long-term users were recruited. Those users could be resistant to some of ayahuasca’s adverse effects, allowing them to use it without perceiving any harm, and thus they tend to participate as volunteers in the studies. However, by analyzing the experience of first-time users, this bias may be better controlled for and more accurate information about the overall effects of ayahuasca on novice users can be obtained.

There are some studies that have focused on the assessment of first-time ayahuasca users. Osório *et al*.^14^ and Sanches *et al*.^15^ reported the effects of a single dose of ayahuasca in patients with recurrent depression in an open-label study. Fast-acting antidepressant and anxiolytic effects were reported that persisted after 21 days. Palhano-Fontes *et al*.^16^ published the first randomized, placebo-controlled trial (RCT) in which ayahuasca was assessed for the treatment of severe depression and significantly reduced depressive symptoms. In those clinical trials, although patients were naïve ayahuasca users, there were carefully selected according to strict inclusion and exclusion criteria. Barbosa *et al*.^24^, in a non-controlled study, found significant decreases in psychiatric symptoms after initial experiences with ayahuasca among a non-clinical sample. Barbosa *et al*.^25^ found similar results in a follow-up study of the same sample. No adverse effects were reported, and those who began to use ayahuasca regularly showed improvements in role-emotional and social functioning scores according to the Short Form-36 Health Survey (SF-36). Trichter *et al*.^26^ analyzed potential changes in spirituality (measured with the Peak Experience Profile, the Spiritual Well-being Scale, and the Mysticism Scale as quantitative measures, and an interview as a qualitative measure) after the first ayahuasca experience of 54 people and found that the experience did not result in significant changes.

**Figure 1:**
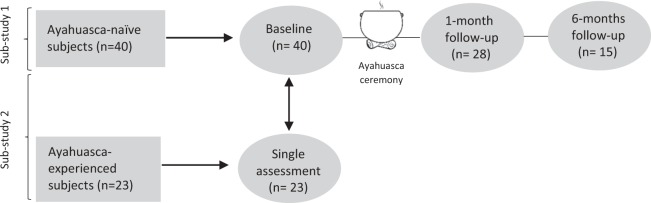
Schematic diagram of the complete study.

Due to our fieldwork, we know that there are dozens of groups of ayahuasca users in the Spanish territory alone, which is a situation that could be relevant to public health^9^. While some authors describe the communitarian use of ayahuasca as a healthcare system^27^, some case reports are being published with information about psychotic episodes induced by ayahuasca^28,29^. When elucidating the potential risks of the ritualistic and communitarian use of ayahuasca, a RCT approach,, would not fit well with the context and the complex variables involved in such ritualistic settings. However, observational studies represent a useful and valuable tool for gathering more generalizable data. In this case, we designed two observational sub-studies: In sub-study 1, a longitudinal assessment of first time ayahuasca users was conducted. Since it is difficult to find a control group/treatment/condition in order to compare results, we designed a sub-study 2, where the baseline assessment of that same group was compared with a group of long-term ayahuasca users. In this way, it is possible to find potential differences that may be attributed to the regular use of ayahuasca or, on the contrary, are common conditions that may explain personal traits that may lead one to get involved in regular ayahuasca use and other spiritual and/or alternative health practices. See Fig. 1 for a schematic diagram of the study.

## Results

For sub-study 1, we recruited 28 women (70%) and 12 men aged between 20 and 65 years ($\overline{X}$ = 35). The majority (95%) of subjects were from Spain. Regarding education, the majority (54%) of the sample graduated from university. Most participants were atheists (84%). The majority of the sample (59.2%) wanted to take ayahuasca for therapeutic purposes, mentioning emotional or psychological issues. Other motivations included self-exploration (29.6%), curiosity (11.1%), to have a psychedelic experience (11.1%), to receive insights regarding professional development (7.4%), or to start on a spiritual path (3.7%). Regarding the types of ceremonies that participants attended, 40% were neoshamanic (led by westerners who traveled to a South American country to learn the methods of traditional medicine), 37.5% religious (mainly Santo Daime, but also other religious groups derived from classical ayahuasca religions), and 22.5% psychotherapeutic (a typical Western approach, in which a psychotherapeutic setting is used, without religious or shamanic content) ceremonies. As is common in longitudinal studies, a smaller number of subjects was retained during follow-ups. While 28 subjects participated in the 1-month follow-up, 15 subjects participated in the 6-months follow-up. At baseline, eighteen subjects (45%; 77.7% women) met the criteria for one or more psychiatric disorders (50% with comorbidity). The most prevalent diagnoses were generalized anxiety disorder (GAD; n = 6) and substance abuse/dependence (alcohol = 2; cannabis = 2; lorazepam = 1). See Table 1.

**Table 1:** Sociodemographic data of the sample.

	Sub-study 1		Sub-study 2	
	n	%	n	%
Gender				
Men	12	30%	13	57%
Women	28	70%	10	43%
Age				
<25	4	10%	—	—
25–35	18	45%	2	9%
36–45	14	35%	14	61%
46–55	2	5%	4	17%
56–65	1	2.50%	3	13%
>65	1	2.50%	—	—
Education				
Basic education	2	5%	3	14%
High-school	6	15%	6	27%
Technical school	3	7.50%	2	9%
University	21	52.50%	8	32%
Doctoral degree	8	20%	4	18%
Nationality				
Spain	38	95%	18	78%
Morocco	1	2.50%	—	—
Venezuela	1	2.50%	—	—
Italy	—	—	2	8.7%
Peru	—	—	1	4.3%
Uruguay	—	—	1	4.3%
Portugal	—	—	1	4.3%
Religious beliefs				
Atheists	34	85%	8	34.8%
Buddhism	2	5%	2	8.7%
Catholicism	2	5%	1	4.3%
Shamanism	1	2.5%	—	—
Taoism	1	2.5%	—	—
Santo Daime			3	13%
MINI interview				
GAD	6	15%	1	4.3%
Substance abuse			—	—
Alcohol	2	5%		
Cannabis	2	5%		
Lorazepam	1	2.5%		
Suicide risk	5	12.5%	2	8.7%
MD	4	10%	—	—
Dysthymia	3	7.5%	—	—
Past Hypomanic	3	7.5%	—	—
episode				
Past manic episode	2	5%	—	—
OCD	3	7.5%	1	4.3%
Antisocial personality	3	7.5%	1	4.3%
disorder				
Social phobia	1	2.5%	—	—
Bulimia	1	2.5%	—	—

GAD = Generalized Anxiety Disorder; MD = Major depression; OCD = Obsessive-Compulsive Disorder.

At the one-month follow-up, the 18 subjects (77.7% women) who met diagnostic criteria were interviewed again. Eleven of them (61%) no longer met the criteria for any psychiatric disorder (0% of comorbidity). Four of these subjects (22.2%) presented fewer psychiatric diagnoses than at baseline. One subject (5.5%) met the criteria for a different psychiatric diagnosis than at baseline. Finally, one subject (5.5%) who did not meet the criteria for any psychiatric disorder at baseline did so during the follow-up, meeting the criteria for GAD. Thus, 7 subjects met the criteria for psychiatric diagnosis at this time-point. One subject had taken ayahuasca once more since the first assessment.

At 6-months follow-up, 15 subjects (60% women) continued their participation in the study. Five subjects took ayahuasca again since the 1-month follow-up. Only 8 subjects from the initial 18 who met the criteria for a psychiatric disorder were interviewed. Among those 18 subjects, only 2 still met the criteria for psychiatric diagnosis. According to the available information, the subjects who met diagnostic criteria at baseline but did not at the 1-month follow-up continued to not present diagnostic criteria for any psychiatric disorder. The participant who did not meet the diagnostic criteria for a psychiatric disorder at baseline but met the criteria for GAD at 1-month follow-up still met the diagnostic criteria for GAD at 6-months follow-up. No other subjects met the criteria for a psychiatric disorder, as only 3 subjects met the criteria at the 6-months follow-up.

Regarding the data from the administered questionnaires, at one-month follow-up, lower scores were found for two scales of the Symptom Check-List-90-Revised (SCL-90-R): anxiety (*d* = 0.54), and hostility (*d* = 0.62). At 6-months follow-up, lower scores were obtained for the Hamilton Depression Rating Scale (HAM-D) (*d* = 0.72) and in the role-emotional scale (*d* = 0.74) from the SF-36 questionnaire. See Table 2.

**Table 2:** Means and confidence intervals obtained in sub-study 1.

HAM-D	Baseline (n = 40)	1-month follow-up (n = 26;)	6-months follow-up (n = 15)
	3.60 [1.77–5.43]	1.75 [0.38–3.12]	0.60 [0–1.35]
CAPE			
Total frequency score	63.3 [59.6–67]	56.2 [49.4–63]	62 [56–68]
Total distress score	39.2 [31.6–46.9]	35.6 [27.9–43.3]	37.1 [23.2–51]
Positive symptoms frequency	26.8 [25.1–28.4]	26 [24.7–27.4]	26.9 [23.8–30]
Positive symptoms distress	10 [7.4–12.6]	8.2 [6.4–10.1]	9.4 [4.7–14.2]
Depression symptoms frequency	13.5 [12.5–14.4]	12.7 [12–13.5]	12.8 [11.3–14.2]
Depression symptoms distress	11.5 [9.3–13.5]	11.2 [8.7–13.6]	10.8 [6.8–14.9]
Negative symptoms frequency	23 [21.4–24.7]	21.5 [19.9–23.1]	22.2 [20.3–24.1]
Negative symptoms distress	17.7 [14.2–21.2]	16.2 [11.8–20.5]	16.8 [10.7–22.8]
SCL-90-R SOM	0.57 [0.39–0.75]	0.45 [0.34–0.57]	0.91 [0.50–1.32]
O-C	0.63 [0.43–0.84]	0.44 [0.24–0.64]	0.82 [0.42–1.21]
I-S	0.60 [0.43–0.78]	0.41 [0.22–0.61]	0.67 [0.25–1.08]
DEP	0.58 [0.38–0.77]	0.40 [0.21–0.60]	0.70 [0.36–1.04]
ANX	0.42 [0.28–0.56]	0.24 [0.17–0.31]	0.58 [0.30–0.85]
HOS	0.52 [0.32–0.73]	0.22 [0.12–0.31]	0.49 [0.23–0.75]
PHOB	0.07 [0.01–0.13]	0.10 [0.03–0.17]	0.32 [0.1–0.54]
PAR	0.40 [0.26–0.54]	0.35 [0.21–0.49]	0.65 [0.22–1.07]
PSY	0.23 [0.12–0.33]	0.13 [0.07–0.19]	0.42 [0.11–0.73]
GSI	0.48 [0.34–0.62]	0.33 [0.22–0.44]	0.64 [0.34–0.93]
PSDI	28.3 [22.1–34.5]	23.51 [17.7–29.3]	42.5 [25.1–60]
PST	1.35 [1.21–1.5]	1.17 [1–1.28]	1.26 [1.07–1.45]
AAQ-II	19.9 [17.3–22.6]	18.2 [15.5–20.8]	18.8 [15.6–22.1]
TCI-R-67 NS	22.4 [20.8–24.1]	21.8 [20.1–23.5]	22.4 [19.2–25.7]
HA	18.7 [17–20.5]	16.6 [15.2–18.1]	17.7 [16.3–19.1]
RD	29.2 [26.9–31.4]	30.6 [29–32.2]	30.5 [27.7–33.3]
PER	25.3 [23.8–26.9]	22.5 [20.3–24.8]	24.2 [22–26.5]
SELFD	30 [28.8–32.4]	32.3 [30.5–34.2]	31.2 [29.1–33.3]
COOP	32 [30.6–33.4]	32 [30–34]	31.8 [29.5–34.1]
ST	20.7 [18.4–23]	21.4 [17.6–25.1]	23.2 [17.8–28.7]
SF-36 PF	97.7 [96–99.5]	98.4 [97.2–99.7]	97.9 [95.8–100]
RP	87.5 [78.2–96.7]	96.1 [88.2–104]	87.8 [72.3–103.3]
BP	78 [69.7–86.2]	89.8 [81.7–97.8]	84.6 [70.9–98.3]
GH	77.5 [71.9–83]	82.5 [77.5–87.5]	72.4 [64.1–80.8]
VIT	62 [55.6–68.3]	70 [64.5–75.4]	70.7 [62–79.4]
SF	81.2 [74–88.4]	88.8 [81.2–96.5]	89.1 [79–99.2]
RE	72.1 [59.3–84.8]	83.3 [70–96.6]	94 [87.1–101]
MH	69.3 [63.6–75]	74.6 [69.8–79.4]	74.4 [65.6–83.1]
WHOQOL-Bref	17.4 [15.1–19.6]	20.67 [17.9–23.4]	21.4 [18.1–24.7]

HAM-D = Hamilton Depression Rating Scale; SCL-90-R = Symptom Check-List-90-Revised; SOM = Somatizations; O-C = Obsessive-Compulsive; I-S = Interpersonal Sensitivity; DEP = Depression; ANX = Anxiety; HOS = Hostility; PHOB = Phobic anxiety; PAR = Paranoid ideation; PSY = Psychoticism; GSI = General Severity Index; PSDI = Positive Symptoms Distress Index; PST = Positive Symptoms Total; AAQ-II = Acceptance and Action Questionnaire; TCI-R-67 = Temperament and Character Inventory Revised; NS = Novelty seeking; HA = Harm voidance; RD = Reward dependence; PER = Persistence; SELFD = Self-directedness; COOP = Cooperativeness; ST = Self-transcendence; SF-36 = The Medical Outcomes Study 36-item Short-Form; PF = Physical function; RP = Role physical; BP = Bodily pain; GH = Global health; VIT = Vitality; SF = Social function; RE = Role emotional; MH = Mental health; WHOQOL = World Health Organization Quality of Life.

Regarding the subgroup analysis of data from the sample that met criteria for diagnosing psychiatric disorders, all of the CIs overlapped between assessments. However, a tendency was observed in scores obtained by most of the questionnaires, as they decreased significantly (data not shown).

Regarding the analysis of potential differences between subjects who dropped out of the study and those who remained, there were no significant differences in any variables, neither at one-month nor at the 6-months follow-up. However, some tendencies with notable effect sizes were registered. At one-month follow-up, subjects who dropped out of the study tended to obtain lower scores in CAPE-depression symptoms frequency [*t*(35.2) = 2.6, *p* = 0.01; *d* = 0.75] and the depression dimension of SCL-90-R [*t*(37.3) = 2.3, *p* = 0.02; *d* = 0.69].

Sub-study 2 was conducted in order to determine if any differences in assessed variables were due to ayahuasca use or previous conditions. In this sub-study, the baseline data of the sample for sub-study 1 were compared with long-term ayahuasca users’ results for the same variables. We recruited 10 women (43%) and 13 men aged between 32 and 64 years ($\overline{X}$ = 45). The majority (78.3%) of the subjects were from Spain. Regarding education, the majority (32%) of the sample graduated from university. In terms of religious beliefs, most participants were atheists (34.8%). The mean number of ayahuasca ceremonies that participants attended was 70 (ranging from 50 to 100 ceremonies). Only two participants (9%) met the criteria for a psychiatric disorder. One subject met the criteria for suicide risk (for attempted suicide), GAD, and obsessive-compulsive disorder (OCD); the other subject met the criteria for suicide risk (for past attempted suicide) and antisocial personality disorder. See Table 1.

Analyzing data collected through the questionnaires, we found statistically significant differences between long-term and ayahuasca naïve users in the HAM-D score [*t*(53.5) = 2.1, *p* = 0.03; *d* = 0.49], the self-transcendence scale from the Temperament and Character Inventory (TCI-R-67) [*t*(61) = 4.6, *p* < 0.001; *d* = 0.51], and the World Health Organization Quality of Life (WHOQOL-Bref) score [*t*(61) = 4.3, *p* < 0.001; *d* = 0.51]. See Table 3.

**Table 3:** Means and standard deviations from the sub-study 2.

HAM-D	Ayahuasca-naïve users (n = 40)	Long-term ayahuasca users (n = 23)	Ayahuasca-naïve users meeting criteria for psychiatric disorders (n = 18)
	3.60 (5.7)	1.48 (2)*	5.61 (7.2)*
CAPE			
Total frequency score	63.3 (11.5)	63.1 (9.6)	68.6 (13.5)
Total distress score	39.2 (23.8)	32.5 (17.9)	48 (26.7)*
Positive symptoms frequency	26.8 (5.2)	28.5 (5.5)	28.7 (5.8)
Positive symptoms distress	10 (8)	10.5 (7.5)	13 (9)
Depression symptoms frequency	13.5 (2.9)	12.3 (2.2)	14.9 (3.3)*
Depression symptoms distress	11.5 (6.5)	8.4 (5.2)	13.9 (6.9)*
Negative symptoms frequency	23 (5)	22.1 (3.5)	24.9 (6.4)
Negative symptoms distress	17.7 (11)	13.5 (7.1)	21 (12.4)*
SCL-90-R SOM	0.57 (0.56)	0.72 (0.4)	0.69 (0.6)
O-C	0.63 (0.64)	0.62 (0.5)	0.85 (0.8)
I-S	0.60 (0.54)	0.57 (0.5)	0.80 (0.6)
DEP	0.58 (0.61)	0.56 (0.6)	0.81 (0.7)
ANX	0.42 (0.43)	0.38 (0.4)	0.56 (0.5)
HOS	0.52 (0.63)	0.38 (0.6)	0.72 (0.7)
PHOB	0.07 (0.18)	0.18 (0.2)	0.06 (0.1)
PAR	0.40 (0.43)	0.60 (0.6)	0.54 (0.4)
PSY	0.23 (0.33)	0.35 (0.4)	0.30 (0.3)
GSI	0.48 (0.43)	0.51 (0.4)	0.63 (0.4)
PSDI	28.3 (19.3)	33 (18.8)	34 (19.4)
PST	1.35 (0.44)	1.23 (0.3)	1.50 (0.5)
AAQ-II	19.9 (8.3)	18.6 (6.3)	23.9 (8.6)*
TCI-R-67 NS	22.4 (5.1)	22 (3.8)	21.7 (4.6)
HA	18.7 (5.5)	17.3 (6.3)	20.1 (6.2)
RD	29.2 (7)	28.8 (6.3)	29.6 (6.8)
PER	25.3 (4.7)	27.5 (4.4)	26.1 (6)
SELFD	30 (5.6)	23.1 (5.6)	29.2 (6.3)
COOP	32 (4.3)	31.8 (4.9)	31.4 (4.4)
ST	20.7 (7.1)	29.7 (7.9)**	21.2 (7.4)*
SF-36 PF	97.7 (5.4)	96.5 (5.5)	97.7 (6.2)
RP	87.5 (29)	91.3 (17.8)	89.5 (30.4)
BP	78 (25.8)	80.4 (21.5)	74.4 (26.1)
GH	77.5 (17.3)	70.8 (13.8)	76.6 (20.9)
VIT	62 (19.8)	62.1 (16.2)	56.9 (19.4)
SF	81.2 (22.4)	87.5 (20)	75 (24.2)
RE	72.1 (40)	89.8 (29)	61.1 (41.6)*
MH	69.3 (17.6)	73.7 (19)	62.9 (19.6)
WHOQOL-Bref	17.4 (7)	24.9 (5.5)**	17.2 (8.1)*

The middle column shows the differences observed between ayahuasca-naïve subjects and long-term ayahuasca users. The column on the right shows the differences found between long-term users and the ayahuasca-naïve participants who met the criteria for diagnosing psychiatric disorders.

HAM-D = Hamilton Depression Rating Scale; SCL-90-R = Symptom Check-List-90-Revised; SOM = Somatizations; O-C = Obsessive-Compulsive; I-S = Interpersonal Sensitivity; DEP = Depression; ANX = Anxiety; HOS = Hostility; PHOB = Phobic anxiety; PAR = Paranoid ideation; PSY = Psychoticism; GSI = General Severity Index; PSDI = Positive Symptoms Distress Index; PST = Positive Symptoms Total; AAQ-II = Acceptance and Action Questionnaire; TCI-R-67 = Temperament and Character Inventory Revised; NS = Novelty seeking; HA = Harm voidance; RD = Reward dependence; PER = Persistence; SELFD = Self-directedness; COOP = Cooperativeness; ST = Self-transcendence; SF-36 = The Medical Outcomes Study 36-item Short-Form; PF = Physical function; RP = Role physical; BP = Bodily pain; GH = Global health; VIT = Vitality; SF = Social function; RE = Role emotional; MH = Mental health; WHOQOL = World Health Organization Quality of Life. * = < 0.05; ** = < 0.001.

Regarding the subgroup analysis involving only the sub-study 1 participants who met the criteria for a psychiatric disorder, more differences were found between those participants and the sample of long-term ayahuasca users. Differences were found in HAM-D score [*t*(39) = 2.6, *p* = 0.03; *d* = 0.77], CAPE-total distress score [*t*(39) = 2.2, *p* = 0.03; *d* = 0.68], CAPE-depression symptoms frequency [*t*(39) = 2.8, *p* = 0.007; *d* = 0.87], CAPE-depression symptoms distress [*t*(39) = 2.9, *p* = 0.006; *d* = 0.89], CAPE-negative symptoms distress [*t*(39) = 2.4, *p* = 0.01; *d* = 0.74], Acceptance and Action Questionnaire (AAQ-II) score [*t*(39) = 2.3, *p* = 0.02; *d* = 0.70], ST scale from TCI-R-67 [*t*(39) = −3.5, *p* = 0.001; *d* = 1.10], RE scale from SF-36 [*t*(39) = −2.5, *p* = 0.01; *d* = 0.79], and WHOQOL-Bref score [*t*(39) = −3.6, *p* = 0.001; *d* = 1.10]. See Table 3.

## Discussion

Since ayahuasca use is expanding both internationally and locally in the Spanish context, the aim of this study was to observe the effects of ayahuasca on psychological and health variables in naïve ayahuasca users. This will help medical practitioners to understand possible adverse effects as well as potential therapeutic uses.

We performed two sub-studies. Regarding sub-study 1, 40 ayahuasca-naïve subjects were assessed before using ayahuasca for the first time, and they were followed up with at 1 and 6 months after. Following the usual pattern of participants in complementary and alternative medicines^30^, the majority of the sample consisted of women with higher education.

The Mini-International Neuropsychiatric Interview (MINI) interview showed that 45% of the sample met the diagnostic criteria for a psychiatric disorder. This finding is in line with the main reasons reported by participants for attending ayahuasca ceremonies, which include the treatment of mental health problems and to achieve psychological well-being^27,31,32^. Remarkably, at the 1-month follow-up, 61% of participants who initially met the diagnostic criteria no longer met the criteria for any psychiatric disorder. Additionally, 22.2% of participants showed a decrease in the number of psychiatric disorders for which they met the diagnostic criteria, reducing the high prevalence of comorbidity at baseline (50%) to 0% at the 1-month follow-up. Overall, 83.2% of participants reported a clinical improvement. This improvement lasted until the 6-months follow-up. This finding is surprising, considering that this clinical improvement reported in the psychiatric interviews was not fully identified by the questionnaires used. A reduction in psychiatric diagnoses based on clinical interviews after the initiation of ayahuasca use is consistent across studies^6,33^. These results are also consistent with a recent study published by our group where it was found that half of a large sample (n = 380) of long-term ayahuasca users reduced or eliminated their prescription drugs after they began to regularly use ayahuasca^9^.

Only one subject met some criteria for a new psychiatric diagnostic, specifically for GAD, after using ayahuasca for the first time. That subject met the criteria for this disorder at 6-months follow-up. There are several reports regarding adverse effects of ayahuasca, leading some of them to psychiatric diagnoses^29^. In order to obtain more information, the questionnaire scores for this case were checked, and it was noted that the scores on every psychopathology scale from the SCL-90-R decreased notably at 1-month follow-up. At 6-months follow-up, half of the scales of the SCL-90-R increased again, following the general trend of the whole sample. In the clinical interview conducted using the MINI, this subject mentioned that the ayahuasca experience went well, and the traumatic termination of a partnership that they were experiencing was a more probable cause of the anxious state.

**Figure 2:**
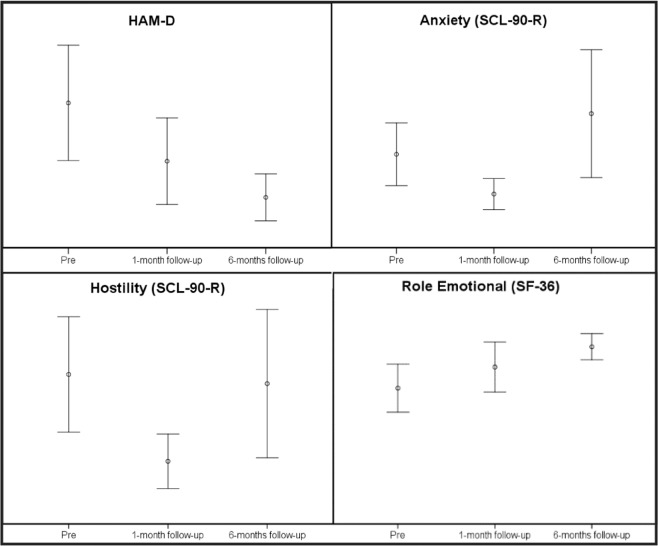
Confidence Intervals showing significant differences through different assessments in sub-study 1. HAM-D = Hamilton Depression Rating Scale; SCL-90-R = Symptom Check-List-90-Revised; SF-36 = The Medical Outcomes Study 36-item Short-Form.

Regarding changes in psychological and psychopathological variables, there were improvements in the HAM-D at the 6-months assessment, in anxiety and hostility from the SCL-90-R at the 1-month assessment, and in the role-emotional scale from the SF-36 (see Fig. 2). The only variable that showed consistent change in the 6-month study period was depression as measured by HAM-D, which improved at every assessment. Although differences between baseline and the 1-month follow-up did not reach statistical significance, the scores decreased by half. This improvement was more evident at the 6-months follow-up, where differences between measures reached statistical significance. This finding is in line with previous research in which ayahuasca showed antidepressant effects at 7 and 21 days after its controlled administration in a clinical setting^14–16^. Our sample was not clinical, but 45% of subjects met the criteria for a psychiatric disorder. The same pattern of improvement was observed in the role-emotional scale from the SF-36. A similar study with a sample similar to ours also found long-term improvements in the role-emotional and social function scales from the SF-36 questionnaire^25^.

Psychopathology scales of anxiety and hostility from the SCL-90-R only improved at 1-month, but not at the 6-months assessment. This tendency was observed for many scales, despite not being statistically significant. A general trend of improvement at 1-month follow-up was observed, while this improvement progressively decreased up until the 6-months follow-up. This can be clearly seen in Table 3. This pattern suggests that the potential therapeutic benefits of ayahuasca are temporary and do not persist in time, excepting for the case of depression. Several studies comparing ritual long-term ayahuasca users with non-users or with normative data have found better SCL-90-R scores and better scores for other psychopathological and psychological measures in users^12,13,33,34^. This may suggest that the long-term benefits of ayahuasca use depend on maintaining regular use.

The size of the sample decreased at both follow-ups. Therefore, we wanted to understand any potential limitations in that regard, so we conducted comparisons between drop-out subjects and those who remained in the study. Despite not obtaining significant results, subjects who scored lower on depression symptoms frequency (CAPE), and depression and hostility (SCL-90-R) at baseline tended to remain as study participants at least until the first follow-up. There were no significant differences nor notable tendencies between subjects who quit and those who remained until the last follow-up regarding any variable. This finding suggests that people with a high degree of distress may feel better after ayahuasca use and, subsequently, be more motivated to collaborate with researchers.

When only participants who met diagnostic criteria for a psychiatric disorder were analyzed, all the confidence intervals (CI) of variables overlapped between assessments. However, they indicated remarkable decrease over time in HAM-D scores, in the psychopathology scores measured by SCL-90-R at the 1-month follow-up, decrease in AAQ-II scores at each assessment, and increase of the WHOQOL-Bref score in the same manner. This also suggests an improvement in psychopathological status as it was also found using the MINI, but the sample may have been too small to observe significant differences using CIs. Despite reporting a clear tendency on the questionnaires used, this was not enough so that the CIs did not overlap.

In order to better understand if the eventual differences found between assessments in sub-study 1 were attributable to the use of ayahuasca or to extrapharmacological variables, including the passing of time, or if there are any pre-conditions that may lead a person to get involved in ayahuasca use, we performed sub-study 2. Only the measure of depression, assessed with the HAM-D, showed significant differences between non-users and long-term users, where the latter scored better than the former, a finding consistent with the long-term antidepressant effects of ayahuasca^14–16^. **In previous studies**, the acute administration of ayahuasca reduced the functional connectivity (FC) of the default mode network (DMN)^21^, which could be related to the improvements made on a depressed condition^35^. **Moreover**, it has been observed that the acute administration of the psychedelic tryptamine psilocybin also reduced the FC in the DMN in patients with MDD^36^ and resulted in a decrease in depression scores that lasted for at least 6 months^37^. Therefore, both the lower scores found at months 1 and 6 in naïve users and the lower scores for depression found in our long-term users, as compared with the baseline scores of subjects from sub-study 1, may reflect an actual mid-term antidepressant effect of ayahuasca. Due to the complex effects of ayahuasca, both pharmacological and psychological, employing an integrated approach involving different disciplines would be necessary to explain its efficacy. It has been observed, for example, that ayahuasca acutely enhances mindfulness-related capacities, such as decentering or acceptance^38,39^, and psychological process variables such as personal values could also be involved in therapeutic outcomes^34^. These capacities seem to be maintained during the after-glow period and also have persistent effects over the long-term^40^. Long-term effects may also be associated with the integration of the experience into normal life (e.g. by reconciling unexpressed/unresolved emotional energy), and thus with improvements in psychotherapeutic outcomes^41^. Furthermore, some authors suggest that the psychedelic experience^42^ or the mystical-type experiences^43^ that psychedelic drugs induce can also explain their therapeutic effects. Finally, as ayahuasca is generally taken in ceremonies for which small or large groups of people gather^44^, its communal use can also be viewed as exerting potential benefits, since feeling part of a community can have an important therapeutic impact^10^.

The other measure where experienced users scored higher than naïve users was self-transcendence (ST). There are multiple studies that have shown that psychedelics can induce long-term personality changes^45^. In sub-study 1, subjects did not change their scores in ST between assessments, but in sub-study 2 long-term users scored significantly higher than non-users in ST, a result that has been consistently found in previous studies as well^11,12,24^. It has been suggested that ayahuasca users’ higher ST scores could be partially explained by their participation in religious practices such as Santo Daime. However, in the study by Bouso *et al*.^12^ the samples (ayahuasca users and non-users) were matched for religion, age, and gender, so the difference in that study could be attributed to the ritualistic use of ayahuasca. In the present study, the number of atheists was lower in the group of long-term ayahuasca users, but only 13% of participants were Santo Daime members. Taking all these data into account, it is reasonable to think that higher scores in ST may be a direct consequence of ayahuasca use. A previous study found differences in cortical thickness in several brain areas to exist between long-term users and non-users of ayahuasca, suggesting a physical brain modification after regular ayahuasca exposure without any apparent neuropsychiatric consequences (there were no differences between groups in their performance on psychiatric and neuropsychological tasks)^13^. In that study, researchers found a correlation between ST and cortical thickness in the posterior cingulate cortex^13^. Psychedelic drugs may therefore offer an interesting tool for investigating the stability of personality traits and their brain correlates.

Regarding the differences between naïve ayahuasca users’ and experienced users’ baseline scores for quality of life as assessed by the WHOQOL-Bref, they are also consistent between sub-studies. Although in sub-study 1 there were no significant differences between assessments, there was an actual clinical improvement between them (see Table 3). This is consistent with previous research where regular ayahuasca use was associated with a better mental health status or with improvement on psychopathology scales, as was also observed in sub-study 1.

When comparing long-term ayahuasca users with the sub-study 1 subsample of psychiatric diagnosis (n = 18), the number of variables in which significant differences are found increases. This is reasonable in light of the much smaller number of long-term ayahuasca users who met the diagnostic criteria for at least one psychiatric disorder (9%). Ayahuasca-naïve users who met the criteria for a psychiatric diagnosis scored poorly for depression (HAM-D), positive symptoms frequency (CAPE), depression symptoms distress (CAPE), negative symptoms distress, in AAQ-II questionnaire, self-transcendence scale (TCI-R-67), role-emotional (SF-36), and WHOQOL-Bref. Despite not finding statistically significant differences between assessments among the subgroup of naïve users with psychiatric disorders, remarkable improvements were observed for several scales measuring psychopathology and quality of life. This lends support to previous research that found that ayahuasca users gained therapeutic benefits or experienced an enhanced ability to solve personal problems^25,33,46^.

The main limitation of this study is that there was no control group with which to make comparisons to determine the impact of the intervention. In order to minimize that limitation, we designed sub-study 2. Coherent results were found both between sub-studies and with previously published research. However, further studies using larger samples should consider potential differences between the type and setting of the ceremony.

This study assessed naïve users of ayahuasca in order to collect data regarding its overall effects on psychiatric condition and psychological status. Since ayahuasca use has become globally popular as a self-help practice, it is important to better understand its potential risks and benefits. General mid-term adverse effects were not observed in this study, although some secondary acute reactions were observed in some individual cases (e.g. anxiety) and will be reported in a separate paper. Additionally, from a clinical point of view, there was a substantial decrease in psychiatric symptomatology after the first use of ayahuasca, which persisted until the 6-months follow-up. The most evident improvements were found with regards to depression. These improvements in depression found after performing clinical interviews were also demonstrated by the psychiatric rating scales. Better scores for depression were also observed among long-term users when compared with ayahuasca-naïve users at baseline. The results obtained in this study help to inform us about the effects of the ritualistic use of ayahuasca on mental health. These results suggest that the use of ayahuasca in controlled settings may offer therapeutic benefits. Future studies with larger samples are warranted in order to better understand 1) the more specific effects of ayahuasca use on public health and 2) the reasons why those initiated in ayahuasca use continue to use it or not.

## Methods

### Sub-study 1

#### Sample

Several ayahuasca ceremony leaders from different parts of Spain were contacted and asked to inform us when ayahuasca-naïve subjects called them to participate in their ayahuasca ceremonies. Subjects willing to participate contacted the authors. In this way we recruited 40 ayahuasca-naïve subjects (28 women) for sub-study 1.

### Measures

Sociodemographic information. Age (years), gender (male/female), nationality, maximum educational level, religion, and motivation for participating in ayahuasca ceremonies were collected from participants.

Mini-International Neuropsychiatric Interview (MINI; Sheehan *et al*.^47^; Spanish version: Ferrando *et al*.^48^). MINI is a short structured diagnostic interview that includes DSM-IV and ICD-10 psychiatric disorders. It is a short but accurate tool for use in both clinical trials and epidemiological studies.

Hamilton Depression Rating Scale (HAM-D; Hamilton^49^; Spanish version: Ramos-Brieva^50^). HAM-D is a questionnaire used to rate the severity of depression through the assessment of various aspects, such as mood, suicide ideation, insomnia, anxiety, or somatic symptoms. A score of 0 to 7 is considered to be normal, while a score of >7 is indicative of depression.

Community Assessment of Psychic Experience (CAPE; Stefanis *et al*.^51^; Spanish version: Fonseca-Pedrero *et al*.^52^). CAPE was designed specifically to evaluate psychotic-like experiences in epidemiological studies in the general population. It contains 42 items and measures psychotic-like experiences using eight sub-scales (Total Frequency score; Total Distress score; Positive Symptoms Frequency; Positive Symptoms Distress; Depression Symptoms Frequency; Depression Symptoms Distress; Negative Symptoms Frequency; Negative Symptoms Distress) based on a dimensional approach.

The Symptom Check-List-90-Revised (SCL-90-R; Derogatis^53^; Spanish version: González de Rivera *et al*.^54^). The SCL-90-R is a self-report questionnaire that assesses 9 psychopathological symptomatic dimensions. It includes 90 Likert-type items that are scored from 0 to 4: Somatization (SOM), Obsessive-Compulsive (O–C), Interpersonal Sensitivity (I–S), Depression (DEP), Anxiety (ANX), Hostility (HOS), Phobic Anxiety (PHOB), Paranoid Ideation (PAR), and Psychoticism (PSY). The scale also provides 3 additional psychopathological indices: General Severity Index (GSI), Positive Symptoms Distress Index (PSDI), and Positive Symptoms Total (PST). For all of these scales, higher scores imply worse symptomatology.

Acceptance and Action Questionnaire (AAQ-II; Bond *et al*.^55^; Spanish version: Ruiz *et al*.^56^). AAQ-II is a measure of psychological flexibility that shows good test–retest reliabilities. High scores imply worse psychological flexibility.

Temperament and Character Inventory (TCI-R-67; Cloninger^57^; Spanish version: Pedrero-Pérez *et al*.^58^). TCI-R-67 is a 67-item self-report questionnaire, in which items are rated between 1 (completely disagree) and 5 (completely agree), that measures seven domains of personality: Novelty Seeking (NS), Harm Avoidance (HA), Reward Dependence (RD), Persistence (PER), Self-directedness (SELFD), Cooperativeness (COOP), and Self-transcendence (ST).

The Medical Outcomes Study 36-item Short-Form (SF-36; Ware & Sharebourne^59^; Spanish version: Alonso *et al*.^60^). SF-36 has been used in many healthcare settings. It has 8 individual subscales divided across physical and psychological domains: Physical Function (PF), Role Physical (RP), Bodily Pain (BP), Global Health (GH), Vitality (VIT), Social Function (SF), Role Emotional (RE), and Mental Health (MH).

World Health Organization Quality of Life (WHOQOL-Bref; WHO^61^; Spanish version: Lucas-Carrasco^62^). WHOQOL-Bref is an abbreviated 26-item version of the WHOQOL-100. It is an assessment tool with cross-cultural validity that is used to assess the quality of life.

### Procedure

The interview and questionnaires were administered before ayahuasca-naïve subjects attended their first ayahuasca ceremony. One-month and 6-months follow-up were conducted in order to observe potential changes in variables assessed.

### Statistical analysis

An analysis based on confidence intervals (CI; 95%) was used. CIs were calculated for each of the variables and assessments. When two CIs of the same variable did not overlap between different assessments, we calculated and reported the effect size (Cohen’s *d*). A subgroup analysis involving only those subjects who met psychiatric diagnostic criteria was conducted.

Two Student’s *t* tests were used to analyze potential differences between those subjects who quit the study at both follow-ups and those who did not. Effect size (Cohen’s *d*) was calculated and reported. Bonferroni correction was used for multiple comparisons. *P* values under 0.001 were considered statistically significant.

IBM SPSS Statistics v.20 was used to analyze the data. An on-line calculator of effect size was used (https://www.uccs.edu/lbecker/).

### Sub-study 2

#### Sample

The same ayahuasca ceremony facilitators were asked to inform us when ayahuasca-experienced subjects (inclusion criterion was established as the participant having taken ayahuasca more than 30 times in their lifetime) called them to participate in their ayahuasca ceremonies. Subjects willing to participate contacted the authors. A total of 23 ayahuasca-experienced subjects (10 women) were recruited.

### Measures

The same measures were used as in sub-study 1.

### Procedure

The interview and questionnaires were administered in one single assessment.

### Statistical analysis

Student’s *t* test was used to compare the means obtained for both samples of sub-study 2. A subgroup analysis was conducted involving only the sub-study 1 subjects who met psychiatric diagnostic criteria and comparing them with the sub-study 2 sample.

IBM SPSS Statistics v.20 was used to analyze the data. An on-line calculator of effect size was used (https://www.uccs.edu/lbecker/).

### Ethics

This study (inclusive of sub-studies 1 and 2) was approved by the Research Ethics Committee of the Universidad Autónoma de Madrid (Autonomous University of Madrid), Spain. Written informed consent was obtained from all volunteers. All experimental procedures were performed in accordance with the relevant guidelines and regulations.
